# Sustaining educational and psychological support during protracted war: a regional laboratory model from Ukraine

**DOI:** 10.3389/fpsyg.2026.1939975

**Published:** 2026-08-25

**Authors:** Artem Kyianovskyi

**Affiliations:** Department of Pedagogy and Educational Psychology, Lyceum “School of Humanitarian Labour” of Kherson Regional Council, Kherson, Ukraine

**Keywords:** collective educational adaptation, Education in Emergencies, educational psychology, knowledge mobilization, professional learning communities, protracted war, psychosocial support, Ukraine

## Abstract

Protracted wars create challenges that extend beyond immediate educational disruption and humanitarian response. As crisis conditions become embedded within everyday life, educational communities must continuously adapt to emerging psychological, social, and educational needs affecting children, families, teachers, and schools. While existing research in Education in Emergencies (EiE) and school-based Mental Health and Psychosocial Support (MHPSS) has substantially expanded the understanding of child wellbeing and intervention strategies during crises, considerably less attention has been devoted to the mechanisms through which educational communities sustain support capacity over time. This study examines a regional Educational-Psychological Laboratory operating in the Kherson region, Ukraine, between 2023 and 2026. Rather than evaluating child-level outcomes directly, the study investigates how educational professionals collectively identified emerging wartime challenges as reflected in practitioners' representations, transformed professional concerns into reported strategies and practical recommendations, and redistributed knowledge through educational networks. A longitudinal qualitative case study design, supplemented by descriptive quantitative indicators, was employed. The empirical corpus included official documents, annual plans, seminar programmes, transcripts of open laboratory sessions, methodological materials, participant questions, consultations, and available feedback generated through laboratory activities. The laboratory functioned as a regional support structure involving approximately 110 educational institutions and a stable professional community of school psychologists, teachers, social pedagogues, and educational leaders. Analysis revealed four major domains of emerging wartime challenges: psychological stability and emotional wellbeing, educational participation and motivation, digital overload and distance-learning adaptation, and support capacity among educational professionals. Findings indicate that the laboratory functioned not simply as a provider of professional development activities but as a mechanism of sustained professional accompaniment through which practitioner-identified concerns were translated into collective reflection, practical knowledge generation, and contextually adapted educational action. Based on these findings, the article proposes the Collective Educational and Psychological Adaptation Model (CEPAM), conceptualizing adaptation as a cyclical process linking emerging challenges, professional requests, collective interpretation, practical recommendations, implementation, and renewed inquiry. The study contributes to Education in Emergencies, professional learning, and knowledge mobilization research by demonstrating how educational communities can sustain response capacity when war becomes part of everyday educational life.

## Introduction

Over the past two decades, Education in Emergencies (EiE) has evolved from a specialized humanitarian concern into a major field of educational research, policy, and practice. Initially focused on maintaining access to schooling during armed conflict, displacement, and natural disasters, the field has progressively expanded its understanding of the role of education in crisis contexts. Contemporary EiE frameworks increasingly recognize that schools contribute not only to learning continuity but also to protection, social connectedness, psychosocial wellbeing, identity formation, and developmental stability [Burde et al., 2017; Inter-Agency Network for Education in Emergencies, 2024; UN Children's Fund (UNICEF), 2024].

This broader understanding has been reinforced by growing international attention to Mental Health and Psychosocial Support (MHPSS) within educational settings. International organizations, including UNICEF, UNESCO, Education Cannot Wait, and the Inter-Agency Network for Education in Emergencies, increasingly emphasize that psychosocial support should not be viewed exclusively as a clinical service delivered outside educational environments. Rather, schools themselves are understood as critical sites for promoting participation, belonging, emotional security, resilience, and developmental continuity among children affected by crisis [Education Cannot Wait, 2022; UN Children's Fund (UNICEF), 2024]. As a result, educational institutions are increasingly expected to address both academic and psychosocial dimensions of children's experiences. This expectation has become particularly significant in contexts of prolonged conflict, where schools frequently serve as one of the few remaining stable social institutions within children's lives (Nicolai, 2003). By addressing these protracted challenges, this study directly aligns with the United Nations Sustainable Development Goals—specifically SDG 3 (Good Health and Wellbeing) and SDG 4 (Quality Education)—by demonstrating how localized, resilient systems can sustain school-based Mental Health and Psychosocial Support (MHPSS) under active, prolonged conflict, where traditional top-down intervention models often face operational limits.

Recent research has substantially expanded the understanding of children's needs during emergencies. Studies have examined trauma-informed education, resilience-building approaches, school-based psychosocial interventions, teacher support, and strategies for maintaining educational participation under conditions of disruption (Maynard et al., 2019; Thomas et al., 2019; Tol et al., 2023). Collectively, this literature has demonstrated that educational environments can play an important role in mitigating the effects of adversity and supporting adaptation during periods of instability. This theoretical foundation is further strengthened by a rapidly growing body of empirical research conducted within Ukraine during the ongoing full-scale war. Recent studies have documented how Ukrainian educators and school psychologists navigate extreme operational stress, adapt psychosocial support models, and foster classroom emotional security amidst continuous disruption (Frazier et al., 2025; Gorbunova et al., 2025; Nadyukova and Frenzel, 2025; Powell et al., 2025). Furthermore, innovative pedagogical methodologies—such as humanitarian art pedagogy—have offered empirical insights into supporting students' psychological resilience under prolonged military threats (Kyianovskyi and Pozdniakova, 2026).

However, while these contributions significantly advance our understanding of crisis response, providing sustained support during protracted conflict poses unique psychological challenges for educational professionals themselves. Rather than operating as neutral external agents, teachers and school psychologists function as “wounded healers”—navigating a shared traumatic reality where they experience the same systemic threats, missile attacks, displacement, and ongoing uncertainty as the children and families they support (Atli and Çitil Akyol, 2026; Banit and Merzliakova, 2023). Under these conditions, educational communities must address chronic issues such as ambiguous loss, secondary traumatic stress, and cumulative burnout to sustain their long-term professional accompaniment and response capacity over time (Paryente and Frei-Landau, 2024).

### The problem of sustained capacity in protracted crises

At the same time, an important limitation remains within the existing literature. Much of the current research focuses on interventions, programmes, support models, and child-level outcomes. These studies provide valuable evidence regarding what forms of support may be beneficial and how educational systems can respond during acute phases of crisis. However, considerably less is known about how educational communities themselves sustain support capacity when crisis conditions persist over extended periods of time.

This limitation becomes particularly important in contexts of protracted war. Unlike short-term emergencies, prolonged conflicts transform crisis conditions into a persistent feature of everyday life. Educational disruption becomes chronic rather than temporary. Uncertainty becomes routine rather than exceptional. Crucially, under conditions of protracted war, educational disruption and displacement generate chronic ambiguous loss—where losses are ongoing, unresolved, and characterized by constant structural uncertainty (Paryente and Frei-Landau, 2024). Standard, time-limited MHPSS interventions, which often operate on assumptions of acute trauma followed by stabilization or closure, are structurally inadequate for such conditions. Instead, educational communities require sustained professional accompaniment—a relational, flexible, and iterative process designed to support professionals as they navigate continuous disruption alongside the communities they serve (Paryente and Frei-Landau, 2024). Teachers, school psychologists, educational leaders, parents, and students must therefore adapt not only to individual crisis events but also to the continuing realities created by war (Henderson, 2023; Teacher Task Force, 2019).

Under such circumstances, educational challenges frequently emerge faster than formal systems of guidance, professional development, and institutional support can respond. New educational, psychological, and social concerns appear continuously. Consequently, educational professionals are often required to identify emerging problems, interpret their significance, and collaboratively generate contextually adapted practical responses—supporting children and families under conditions where pre-packaged or standardized interventions are frequently absent or insufficient.

### The Ukrainian context and the role of the laboratory

The war in Ukraine provides a particularly critical context for examining these processes, specifically within high-risk frontline regions such as Kherson Oblast. Since February 2022, educational institutions in Kherson have operated under extreme operational constraints—including continuous artillery and drone bombardment, forced displacement, severe infrastructure damage, prolonged distance learning, severe digital overload, and acute psychosocial distress among both students and educators (United Nations Office for the Coordination of Humanitarian Affairs, 2025; UNESCO, 2024; World Bank, 2022). Educational professionals in this region have been required to address complex, overlapping challenges extending far beyond traditional academic instruction, including emotional stabilization, adaptation to chronic uncertainty, family crisis support, and preserving educational continuity.

Importantly, many of these challenges have evolved over time. During the initial stages of the war, professional concerns frequently focused on safety, acute stress reactions, crisis response, and emergency adaptation. As the conflict continued, however, educational professionals increasingly encountered challenges associated with chronic uncertainty, emotional exhaustion, professional burnout, digital fatigue, declining educational motivation, and the long-term consequences of prolonged disruption (UNESCO, 2025). Traditional MHPSS models and short-term humanitarian interventions, while vital for immediate stabilization, were not structurally designed to provide continuous, long-term support for professionals navigating this evolving reality.

This situation raises an important question for educational research: *How do educational communities sustain educational and psychological support when crisis becomes part of everyday life?* Existing literature provides substantial evidence regarding the educational and psychological consequences of war for children. However, significantly less attention has been devoted to understanding how educational professionals collectively generate practical responses to evolving challenges and how professional knowledge is created, adapted, and redistributed under conditions of prolonged crisis.

The present study addresses this gap through an analysis of a regional Educational-Psychological Laboratory operating in the Kherson region, Ukraine, between 2023 and 2026. Importantly, the laboratory is not treated as the primary object of investigation. Rather, it serves as an empirical window through which broader processes of collective educational and psychological adaptation become visible.

Established by the Department of Education and Science of the Kherson Regional State Administration, the laboratory functioned as a regional framework for professional accompaniment and peer support. It brought together school psychologists, teachers, educational leaders, and social pedagogues to collectively examine emerging challenges, exchange adaptive strategies, and co-design contextualized recommendations for frontline schools (UNESCO, 2023). Rather than operating as a top-down training programme or a short-term intervention project, the laboratory was structured as a continuous, iterative professional space. Through regular online seminars, peer consultations, and methodological roundtables, it sought to facilitate horizontal knowledge exchange and sustain professional capacity under conditions of prolonged conflict.

### Research questions

The study is situated at the intersection of Education in Emergencies, school-based MHPSS, trauma-informed education, professional learning communities, knowledge mobilization, and adaptive educational systems. It examines how educational professionals collectively identified emerging wartime challenges, transformed professional concerns into practical recommendations, and redistributed knowledge through educational networks. The article addresses the following research questions:

1. RQ1. What educational, psychological, and institutional challenges faced by frontline educators were documented through the laboratory's professional activities between 2023 and 2026?
2. RQ2. How did the nature and prioritization of these professional challenges evolve as the acute crisis transitioned into a protracted war?
3. RQ3. Through what collaborative mechanisms were emerging, unstructured wartime concerns translated into practical educational and psychological responses?
4. RQ4. How did participants perceive the role of the laboratory as a mechanism for sustained professional accompaniment, resilience, and mitigation of burnout?
5. RQ5. What conceptual model of collective educational and psychological adaptation under protracted war can be reconstructed from the multi-source empirical corpus?

### Contribution to the literature

The present study contributes to international and comparative education research in three principal fields simultaneously:

First, it extends Education in Emergencies (EiE) scholarship by shifting focus from static crisis outcomes to dynamic, ongoing response capacity in wartime settings. Building upon recent empirical work on Ukrainian frontline education (Frazier et al., 2025; Gorbunova et al., 2025; Nadyukova and Frenzel, 2025), this study offers a longitudinal examination of professional dialogue generated within a regional educational community under continuous conflict. It advances understanding of how frontline practitioners sustain psychosocial support amidst persistent structural disruption and acute operational stress (Burde et al., 2017; Masten and Motti-Stefanidi, 2020; Powell et al., 2025).

Second, it contributes to professional learning and knowledge mobilization research in high-adversity contexts. It highlights how practical, context-specific knowledge is co-constructed when educational professionals act as “wounded healers”—navigating a shared traumatic reality where they experience the same threats, loss, and chronic uncertainty as the populations they serve (Atli and Çitil Akyol, 2026; Banit and Merzliakova, 2023). The study demonstrates how horizontal peer exchange serves as a buffer against secondary traumatic stress and professional burnout, sustaining capacity when centralized guidance cannot adapt at the pace of evolving crisis conditions (Cooper et al., 2009; Levin, 2013; Vescio et al., 2008).

Third, the study introduces the concept of Collective Educational and Psychological Adaptation and proposes the Collective Educational and Psychological Adaptation Model (CEPAM). This framework conceptualizes adaptation under chronic ambiguous loss (Paryente and Frei-Landau, 2024) as an iterative, cyclical process linking emerging challenges, practitioner inquiries, collective interpretation, practical knowledge generation, educational action, and renewed reflection.

By shifting attention from short-term, top-down crisis interventions toward the mechanisms of sustained professional accompaniment, the study offers a complementary, systemic perspective on how educational communities maintain resilience, prevent cumulative burnout, and preserve instructional continuity in contemporary conflict-affected settings.

## Literature review

### Education in Emergencies and the expanding functions of schooling

Research on Education in Emergencies (EiE) has consistently demonstrated that schools perform functions extending far beyond academic instruction during periods of crisis. In contexts of armed conflict, forced displacement, natural disasters, and prolonged instability, educational institutions contribute not only to learning continuity but also to protection, social connectedness, psychosocial wellbeing, and the restoration of daily routines (Burde et al., 2017; Inter-Agency Network for Education in Emergencies, 2024).

This broader understanding reflects a significant shift within international educational policy. Earlier approaches frequently focused on restoring access to schooling after disruption. Contemporary EiE frameworks increasingly recognize that education itself constitutes a protective environment capable of supporting children's emotional, social, and developmental needs. Consequently, schools are expected not only to deliver academic instruction but also to foster belonging, predictability, participation, and hope under continuous adversity [Education Cannot Wait, 2022; Frazier et al., 2025; Nicolai, 2003; UN Children's Fund (UNICEF), 2024].

The Ukrainian context illustrates the critical importance of these expanded educational functions, particularly under persistent military disruption. As war-related disruption shifted from an acute shock to a chronic reality, educational institutions faced conditions of prolonged ambiguous loss—where threat, displacement, and operational uncertainty remain continuously active and unresolved (Paryente and Frei-Landau, 2024). Consequently, schools assumed long-term responsibilities for emotional stabilization, social connection, and developmental continuity (Gorbunova et al., 2025; UNESCO, 2024; World Bank, 2022). This shift challenges standard EiE frameworks, demonstrating that short-term emergency interventions must yield to model frameworks of sustained professional accompaniment capable of supporting educational communities through protracted crisis.

### Mental Health and Psychosocial Support in educational settings

Mental Health and Psychosocial Support (MHPSS) has become a central component of educational responses in humanitarian settings. International organizations increasingly advocate for integrating psychosocial support directly into educational environments rather than treating it as an isolated clinical service delivered outside the school system [Education Cannot Wait, 2022; UN Children's Fund (UNICEF), 2024].

School-based MHPSS frameworks emphasize strengthening protective factors around children, supporting participation in learning, promoting social connectedness, and reducing systemic barriers created by stress, displacement, and acute uncertainty. Educational settings are therefore viewed not merely as physical locations where support can be passively delivered, but as dynamic, protective environments that actively sustain ongoing psychosocial wellbeing under condition of ongoing conflict (Gorbunova et al., 2025; Tol et al., 2023; World Health Organization UNICEF, 2024).

Despite substantial advances in this field, much of the MHPSS literature remains heavily focused on short-term intervention design, discrete programme implementation, and child-level outcomes. Comparatively less attention has been devoted to understanding how educational communities themselves navigate shared trauma—where educational professionals operate as “wounded healers” who simultaneously experience the same chronic threats, loss, and displacement as their students (Atli and Çitil Akyol, 2026; Banit and Merzliakova, 2023). In situations of prolonged war, frontline practitioners frequently encounter complex psychosocial challenges that evolve much faster than institutional support systems can adapt. Consequently, there is an urgent need to shift scholarly focus toward frameworks of sustained professional accompaniment that build continuous peer-support structures, mitigate secondary traumatic stress, and empower educators to co-design practical responses from within their shared reality (Paryente and Frei-Landau, 2024; Powell et al., 2025).

### Trauma-informed education and the need for context-sensitive evidence

Trauma-informed education has become one of the most influential frameworks for understanding children's responses to adversity. The approach encourages educators to interpret behavioral, emotional, and learning difficulties through the lens of traumatic experiences rather than through deficit-oriented assumptions **(**Brunzell et al., 2015; Thomas et al., 2019).

While trauma-informed perspectives have contributed significantly to educational practice by promoting greater sensitivity to children's experiences and encouraging supportive educational environments, the evidence base remains complex. In a major Campbell systematic review, Maynard et al. (2019) concluded that rigorous evidence regarding the effectiveness of school-wide trauma-informed approaches remains limited. While this finding does not diminish the importance of trauma-informed perspectives, it highlights the continuing need for context-sensitive research capable of documenting how educational communities adapt trauma-related knowledge to local realities under conditions of continuous wartime distress (Kyianovskyi and Pozdniakova, 2026; Nadyukova and Frenzel, 2025).

For wartime educational environments, this observation is particularly relevant. Educational professionals navigating chronic ambiguous loss—where trauma is ongoing, unresolved, and shared by educators themselves as “wounded healers”—frequently find standardized, pre-packaged Western trauma protocols ill-suited to frontline conditions (Atli and Çitil Akyol, 2026; Paryente and Frei-Landau, 2024). Consequently, effective practice-generated knowledge relies on frameworks of sustained professional accompaniment that facilitate continuous, horizontal exchange, allowing communities to co-create ecologically valid, context-sensitive evidence grounded in lived educational reality (UNESCO, 2024, 2025).

### Professional Learning Communities and collective adaptation

The concept of Professional Learning Communities (PLCs) emphasizes the collective nature of professional growth and educational improvement. Rather than viewing expertise as an individual characteristic, PLC research highlights the importance of collaboration, shared reflection, collective problem-solving, and continuous professional dialogue (Stoll et al., 2006; Vescio et al., 2008).

Studies suggest that collective reflection strengthens professional capacity, improves decision-making, and facilitates adaptation to changing educational conditions. Through collaborative processes, educational professionals develop shared understandings of complex challenges and create coordinated responses extending beyond individual classrooms and institutions (DuFour, 2004). In high-adversity contexts, peer-led professional communities also function as critical spaces for mitigating secondary traumatic stress, enabling practitioners to navigate shared trauma collectively (Atli and Çitil Akyol, 2026; Banit and Merzliakova, 2023).

However, most PLC scholarship has been developed within relatively stable educational environments. Comparatively less empirical work examines how professional communities maintain structural coherence and emotional sustainability under conditions of chronic warfare, continuous shelling, infrastructure disruption, and prolonged displacement, such as those observed in frontline regions like Kherson Oblast (Frazier et al., 2025; Powell et al., 2025). When institutional systems face severe operational disruption, traditional top-down training gives way to frameworks of sustained professional accompaniment. Within these frameworks, educators operate as “wounded healers” who rely on horizontal, reflective dialogue to co-construct adaptive strategies and sustain institutional continuity amidst ongoing crisis (Paryente and Frei-Landau, 2024; Teacher Task Force, 2019).

### Knowledge mobilization and educational response capacity

Knowledge mobilization research examines how knowledge is generated, shared, adapted, and used within educational systems. Contemporary perspectives challenge traditional assumptions that knowledge flows unidirectionally from academic researchers to practitioners. Instead, knowledge is increasingly understood as a multi-directional phenomenon that emerges through ongoing interaction among researchers, policymakers, practitioners, and local communities (Cooper et al., 2009; Levin, 2013; Ward, 2017).

This perspective is particularly relevant during crises. Educational professionals often encounter novel challenges for which established solutions may be limited or entirely unavailable. Under such circumstances, practitioners—operating as “wounded healers” who actively experience the same chronic threats and operational instability as the populations they serve—become active co-producers of knowledge rather than passive recipients of external expertise (Atli and Çitil Akyol, 2026; Banit and Merzliakova, 2023; Damschroder et al., 2009).

Knowledge mobilization frameworks therefore offer a vital analytical lens for understanding how frontline educational communities mitigate conditions of prolonged ambiguous loss (Paryente and Frei-Landau, 2024). They illuminate the mechanisms through which grassroots concerns transform into collective inquiries, peer-led reflection generates adaptive strategies, and practical solutions are rapidly disseminated across professional networks. In heavily impacted frontline regions such as Kherson Oblast, where top-down systemic guidelines frequently lag behind fast-evolving wartime realities, these horizontal mobilization processes—rooted in frameworks of sustained professional accompaniment—play a decisive role in preserving educational response capacity and preventing operational collapse (Frazier et al., 2025; Inter-Agency Network for Education in Emergencies, 2024; Powell et al., 2025).

### Identification of the theoretical research gap

Current literature provides substantial evidence regarding the educational, social, and psychological consequences of crisis for children, generating important insights into resilience, trauma, psychosocial wellbeing, and educational disruption (Bangpan et al., 2024; Masten, 2001; Ungar, 2011). However, a significant theoretical and empirical gap remains regarding how educational communities navigating chronic ambiguous loss generate, adapt, and mobilize practical knowledge when educators themselves experience shared trauma as “wounded healers” (Atli and Çitil Akyol, 2026; Paryente and Frei-Landau, 2024). This gap is particularly pronounced in frontline zones such as Kherson Oblast, where prolonged military disruption alters standard educational operations.

Most existing studies focus either on static child outcomes or on the short-term evaluation of pre-packaged, externally designed interventions. Far fewer studies investigate the internal mechanisms through which educational professionals collectively identify evolving psychosocial problems, transform localized concerns into actionable recommendations, and sustain educational response capacity over multiple years of active warfare (Gorbunova et al., 2025; Powell et al., 2025). Furthermore, current literature rarely addresses how models of sustained professional accompaniment can replace static, top-down training to support practitioner resilience. This study addresses these theoretical gaps by examining the collective, systemic processes visible within a regional educational network in Kherson Oblast operating under protracted wartime conditions.

## Context and background

### Educational life during protracted war in Ukraine

Since February 2022, the Ukrainian educational system has operated under conditions of prolonged disruption caused by the full-scale Russian invasion. Educational institutions have faced repeated interruptions of learning, wholesale displacement of students and teachers, destruction of infrastructure, mass migration, extended periods of emergency distance education, and compounding psychological pressure affecting children, families, and educators alike (World Bank, 2022). Although many of these challenges initially required immediate responses to an acute crisis, the continuation of the war gradually transformed them into long-term educational realities. Educational communities increasingly confronted not only immediate safety concerns but also problems associated with chronic uncertainty, emotional exhaustion, declining learning motivation, social isolation, digital overload, and systemic developmental disruption.

Under such circumstances, educational institutions were required to perform functions extending far beyond traditional teaching and academic responsibilities. Teachers, school psychologists, educational leaders, and social pedagogues became primary actors in supporting emotional wellbeing, maintaining educational participation, and sustaining developmental continuity. Operating as “wounded healers” navigating shared trauma alongside their students, these professionals assumed expanded psychosocial responsibilities under conditions of ongoing threat [Atli and Çitil Akyol, 2026; Banit and Merzliakova, 2023; UNESCO, 2025; UN Children's Fund (UNICEF), 2024].

The concept of a protracted crisis is particularly relevant in this context. Unlike short-term emergencies, prolonged crises require educational communities to adapt continuously to changing conditions while simultaneously preserving learning, participation, and social support. Educational systems therefore face the core challenge of maintaining educational response capacity over time rather than simply responding to isolated, discrete emergency events.

Kherson Oblast represents a particularly critical and complex setting for examining these adaptive dynamics. The region underwent Russian occupation, active combat, liberation, and subsequent continuous frontline bombardment, characterized by daily artillery shelling and targeted FPV drone attacks on civilian infrastructure. These security realities made in-person schooling impossible, enforcing mandatory emergency distance learning across geographically fragmented communities. Under conditions of prolonged ambiguous loss, educational professionals confronted acute safety risks, infrastructure destruction, displacement, and severe psychological exhaustion (Paryente and Frei-Landau, 2024). Lacking pre-existing methodological protocols for such extreme conditions, local educational communities could not rely on static, short-term interventions. Instead, they required frameworks of sustained professional accompaniment to organically co-design practical, context-sensitive responses that preserved educational continuity amidst active warfare (Frazier et al., 2025; Powell et al., 2025; UNESCO, 2024; United Nations Office for the Coordination of Humanitarian Affairs, 2025).

### Establishment of the regional educational-psychological laboratory

In response to these systemic challenges, the Department of Education and Science of the Kherson Regional State Administration established the Educational-Psychological Laboratory for the Rehabilitation of Participants in the Educational Process under Martial Law by official Order No. 26 on June 28, 2023. The laboratory was created as a centralized regional support structure intended to assist educational institutions operating under wartime conditions. According to its founding documentation, the laboratory was mandated to provide systematic psychological, pedagogical, consultative, methodological, and rehabilitation-oriented support for participants in the educational process under conditions of martial law.

Its establishment reflected a growing recognition that educational communities required systematic opportunities for professional consultation, collective reflection, and practical guidance in response to rapidly changing educational and psychological realities. According to its founding documents, the laboratory was designed to mitigate acute threats to psychological wellbeing through integrated diagnostic, consultative, corrective, and methodological activities. It explicitly addressed the severe operational and psychological realities of Kherson Oblast, where frontline educational staff operated as “wounded healers” navigating shared trauma under continuous military threats (Atli and Çitil Akyol, 2026; Paryente and Frei-Landau, 2024). Its institutional responsibilities included supporting children, teachers, school psychologists, and educational leaders, while facilitating the development and dissemination of practical recommendations relevant to wartime educational conditions.

Importantly, the laboratory was not conceived as a standard academic research project, a clinical therapeutic programme, or a short-term intervention initiative. Instead, it was established as an institutionalized mechanism of sustained professional accompaniment intended to respond dynamically to emerging educational and psychosocial challenges identified directly within frontline practice. This operational design distinguishes the laboratory from externally funded, time-bound training programs that often offer limited longitudinal follow-up. By functioning as an embedded, permanent professional infrastructure, the laboratory facilitated continuous peer accompaniment, horizontal knowledge generation, and long-term psychosocial stabilization across Kherson's educational network (Frazier et al., 2025; Inter-Agency Network for Education in Emergencies, 2024; Powell et al., 2025; UNESCO, 2023).

### Structure, activities, and regional reach

Between 2023 and 2026, the laboratory organized a continuous cycle of professional activities that included seminars, round tables, targeted consultations, methodological discussions, open professional sessions, practical workshops, and the development of actionable recommendations. These professional activities were organized systematically throughout the academic year. On average, the laboratory conducted approximately three major regional seminars annually, regular laboratory meetings devoted to reviewing emerging requests, targeted round tables, and ongoing consultative activities responding to immediate concerns raised by local educational institutions.

Unlike traditional professional development programmes based on rigid, predetermined curricula, the content of laboratory activities emerged primarily from practical, bottom-up requests generated within the educational communities themselves. Inquiries and operational dilemmas raised by school psychologists, teachers, and educational administrators—acting as frontline practitioners navigating shared trauma—served as the direct empirical starting point for reflective discussions, peer support, and subsequent methodological publications within a model of sustained professional accompaniment (Atli and Çitil Akyol, 2026; Levin, 2013; Paryente and Frei-Landau, 2024; Ward, 2017).

Across the 2023–2026 period, the laboratory maintained a systematic regional professional network encompassing approximately 110–112 educational institutions (general secondary schools, lyceums, and preschools) operating across frontline communities in Kherson Oblast. A core cohort of approximately 90–100 practitioners consistently participated in laboratory sessions, while overall attendance varied depending on topic-specific needs. School psychologists constituted the primary participant group, complemented by general educators, social pedagogues, and institutional leaders. This multidisciplinary stakeholder composition provided systematic data triangulation across varying professional roles and educational levels (DuFour, 2004; Stoll et al., 2006). To accommodate geographical fragmentation and active bombardment in Kherson Oblast, activities were conducted asynchronously and synchronously online, with sessions systematically recorded, transcribed, and archived to ensure sustained access (Powell et al., 2025; UNESCO, 2025).

### From intervention to accompaniment

One of the most distinctive features of the laboratory concerned its underlying support philosophy, which marked a paradigm shift from intervention to accompaniment. Many contemporary educational and psychosocial initiatives in crisis contexts are organized around an intervention logic, within which external specialists deliver targeted, bounded activities designed to address specific, pre-defined problems. While these interventions are often effective, they remain limited in duration, sustainability, and scope (Bangpan et al., 2024; Maynard et al., 2019).

The laboratory developed a different, localized model. Rather than relying exclusively on bounded external interventions, it functioned as an institutionalized mechanism of sustained professional accompaniment. Recognizing that educators in Kherson Oblast operated as “wounded healers” experiencing shared trauma alongside their students, this framework provided continuous horizontal spaces for peer consultation, reflection, and co-designing localized coping strategies (Atli and Çitil Akyol, 2026; Paryente and Frei-Landau, 2024). Educational professionals were able to bring raw, emerging concerns directly into collective discussion, receive immediate peer and expert consultation, access systemic support, and participate in the co-creation of practical responses. This iterative process created an environment in which educational communities could continuously identify new challenges and adapt their pedagogical practices in real time (Damschroder et al., 2009; Inter-Agency Network for Education in Emergencies, 2024).

As protracted warfare persisted, practitioner inquiries evolved from acute safety protocols toward addressing chronic ambiguous loss, digital overload, declining student motivation, secondary traumatic stress, and systemic burnout (Gorbunova et al., 2025; Masten and Motti-Stefanidi, 2020). Consequently, the laboratory served as a regional platform for peer-led knowledge mobilization, enabling frontline educational communities in Kherson Oblast to interpret shifting realities and sustain educational continuity amidst prolonged crisis (Frazier et al., 2025; Powell et al., 2025; Tol et al., 2023). Within this study, the laboratory is conceptualized not as a standardized intervention program, but as an empirical locus for analyzing collective professional adaptation under continuous wartime disruption.

## Methods

### Research design

This study employed a longitudinal qualitative case study design, supplemented by descriptive quantitative metrics. The primary objective was not to evaluate the clinical or pedagogical efficacy of a bounded intervention or training programme, but rather to analyze how frontline educational practitioners operating as “wounded healers” collectively interpreted, adapted, and mobilized practical knowledge amidst continuous shared trauma and ambiguous loss (Atli and Çitil Akyol, 2026; Paryente and Frei-Landau, 2024). A case study approach was considered uniquely appropriate because the Educational-Psychological Laboratory represented a naturally occurring, bounded professional environment in which real-world educational actors identified challenges, exchanged localized experiences, generated practical recommendations, and supported educational institutions operating under active wartime conditions (Burde et al., 2017; Tol et al., 2023).

The longitudinal qualitative case study framework combined qualitative thematic analysis of professional interactions with descriptive quantitative metrics tracking participation trends, activity structures, and thematic frequencies across the 2023–2026 period. Qualitative methods enabled the reconstruction of iterative knowledge mobilization and collective coping mechanisms, while descriptive metrics contextualized the regional reach and structural continuity of the laboratory's sustained professional accompaniment framework in Kherson Oblast (Frazier et al., 2025; Powell et al., 2025).

### Case context

The case examined in this study is the Educational-Psychological Laboratory for the Rehabilitation of Participants in the Educational Process under Martial Law, formally established by the Department of Education and Science of the Kherson Regional State Administration in June 2023. The laboratory functioned as a centralized regional support mechanism serving educational institutions across the structurally and geographically fragmented Kherson region. Its operational cycle included a continuous schedule of regional seminars, thematic round tables, structured consultations, collaborative methodological discussions, open professional development sessions, and the collaborative preparation of practical recommendations responding directly to emerging educational and psychological challenges.

Between 2023 and 2026, the laboratory sustained a regional professional network encompassing approximately 110–112 educational institutions (general secondary schools, lyceums, and preschools) operating across frontline communities in Kherson Oblast. A core cohort of 90–100 educational practitioners consistently participated in key laboratory sessions, supplemented by wider thematic attendees. School psychologists comprised the primary participant demographic, working alongside general educators, social pedagogues, and institutional administrators. Operating within a framework of sustained professional accompaniment, this multidisciplinary composition enabled peer support and cross-role data triangulation among professionals navigating shared trauma and continuous ambiguous loss under active frontline conditions (Atli and Çitil Akyol, 2026; DuFour, 2004; Paryente and Frei-Landau, 2024; Powell et al., 2025) (Table 1).

**Table 1:** Scope and structure of laboratory operational activities (2023–2026).

Indicator	Metric/Value
Official institutional basis	Order No. 26 (Department of Education and Science, Kherson RSA, June 28, 2023)
Regional operational scope	Kherson Oblast (frontline distance-learning educational network)
Institutional engagement	110–112 educational institutions (schools, lyceums, preschools)
Core participant cohort	90–100 recurring educational practitioners
Primary professional groups	School psychologists (primary demographic), general educators, social pedagogues, school administrators
Key synchronous events	Eight regional open seminars; five thematic round tables; regular quarterly laboratory meetings
Methodological outputs	Four published regional methodological recommendation packages
Consultative mechanisms	Continuous request-based individual and group professional consultations
Asynchronous dissemination	Secure regional educational repositories, archived session transcripts, and digital video recordings

### Data sources

The empirical dataset analyzed in this study comprises a structured corpus of professional documentation systematically generated through laboratory activities across the 2023–2026 period (*N* = 1,248 analytical text units). In this study, an “analytical text unit” is defined as a discrete, thematic text segment (e.g., a specific practitioner inquiry, a coded transcript excerpt, or a distinct methodological recommendation paragraph) representing a complete professional interaction or concern.

The empirical corpus was categorized into four primary data tracks:

- Regulatory and institutional artifacts: official administrative orders, laboratory founding mandates, annual operational plans, and formal regional progress reports.
- Operational and session materials: seminar agendas, full verbatim transcripts of open professional sessions, structured meeting minutes, and direct documentation of group consultations.
- Practitioner-generated inquiries: written requests, submitted practitioner questions, and anonymous feedback logs provided by school psychologists, general educators, and administrators.
- Methodological outputs and dissemination materials: published regional methodological recommendations, guidance briefs, and archived digital session logs.

Because these materials were derived from authentic professional activities rather than artificial experimental settings, they reflect naturally occurring practitioner communication. This provides direct insights into evolving professional concerns, localized coping mechanisms, and organic knowledge mobilization under conditions of chronic wartime disruption (Burde et al., 2017; Inter-Agency Network for Education in Emergencies, 2024; Powell et al., 2025) (Table 2).

**Table 2:** Composition and structural breakdown of the empirical data corpus (2023–2026).

Data track	Primary material types	Timeframe	Volume/Unit count (*N* = 1,248)	Approx. word count	Selection/Inclusion criteria
Institutional artifacts	Regulatory orders, annual plans, formal activity reports	2023–2026	42 official documents (*n* = 118 units)	~35,000	Official administrative status; relevance to laboratory governance and mandates
Session transcripts and minutes	Full transcripts of open seminars, round tables, and quarterly meetings	2023–2026	17 full session transcripts (*n* = 512 units)	~140,000	Complete audio/textual records of synchronous regional events
Practitioner inquiries and logs	Written questions, consultative request forms, and feedback logs	2023–2026	320 inquiry records (*n* = 436 units)	~65,000	Submitted by active educators/psychologists; containing explicit psychosocial or pedagogical queries
Methodological outputs	Published guidelines, advisory briefs, and summary recommendations	2023–2026	Four published packages (*n* = 182 units)	~45,000	Formally approved regional methodological guidance documents

### Analytical strategy

Data analysis was executed using an abductive, longitudinal qualitative content and thematic analysis approach (Braun and Clarke, 2021; Krippendorff, 2019; Saldaña, 2021). The analysis proceeded through four structured stages:

1. Data familiarization and parsing: the entire corpus (*N* = 1,248 units) was cataloged chronologically across the 2023–2026 period to identify recurring practitioner concerns and track temporal shifts from acute crisis response to sustained adaptation.
2. Initial coding and codebook development: coding combined inductive open coding (capturing raw practitioner language and emerging frontline realities) with deductive orientation toward established crisis education frameworks (e.g., MHPSS, EiE). Coding was performed independently by two primary researchers. Inter-coder reliability was assessed on a randomized 15% sample of the corpus, achieving high initial agreement (kappa = 0.87). Discrepancies were resolved through deliberative consensus meetings involving a third senior researcher.
3. Category refinement and trajectory mapping: initial codes were aggregated into sub-themes and broader conceptual categories. Special analytical attention was paid to tracing how raw practitioner inquiries were transformed through peer consultations into structured methodological outputs (a audit trail demonstrating this data transformation from raw quotes to codes, categories, and CEPAM dimensions is provided in Supplementary Table S1).
4. Model synthesis: the relationships among practitioner inquiries, horizontal accompaniment activities, and organizational outcomes were cross-synthesized to reconstruct the processual dimensions of the CEPAM framework.

### Trustworthiness

To ensure qualitative trustworthiness, credibility, and dependability, several systematic procedures were implemented across all analytical stages. First, data triangulation was established by integrating multiple empirical tracks, contrasting official regulatory artifacts with verbatim session transcripts, practitioner inquiry logs, and published methodological outputs (Damschroder et al., 2009; Thomas et al., 2019). Second, methodological triangulation combined qualitative content and thematic analysis anchored by descriptive participation metrics (Maynard et al., 2019).

Third, an explicit analytical audit trail—encompassing operational codebooks, decision logs, and raw-to-category mapping—was systematically documented to maintain procedural transparency (Supplementary Table S1). Emerging interpretations were continuously cross-checked across different data streams within the corpus. This continuous comparative approach minimized the influence of isolated or atypical examples, ensuring that the findings reflect structural realities consistent across the entire dataset. Priority was placed on contextual credibility, analytical transparency, and transferability rather than statistical generalizability.

## Researcher positionality

To maintain qualitative rigor and reflexive transparency, the researchers' positionality within this case must be articulated. The lead researcher operated as an active practitioner within the regional educational infrastructure of Kherson Oblast and participated in laboratory activities throughout 2023–2026. This “insider” status (practitioner-researcher) facilitated mutual trust and deep contextual comprehension of frontline realities. However, to mitigate potential subjectivity and confirmation bias, strict methodological safeguards were enforced. These included independent double-coding (kappa = 0.87), formal audit sessions with an external senior researcher, and a systematic search for disconfirming evidence (e.g., implementation barriers, negative cases, or unaddressed practitioner inquiries) within the text corpus (Braun and Clarke, 2021; Saldaña, 2021). These procedures ensured that the conceptualization of the CEPAM model remains strictly grounded in empirical textual evidence rather than subjective personal experiences.

### Ethical considerations

This study analyzed administrative documentation and professional communications generated among adult educational practitioners; it involved no direct primary data collection from or interventions with children. All child-related observations within the dataset reflect indirect adult practitioner assessments and pedagogical observations rather than child-level diagnostic metrics. To safeguard institutional and individual confidentiality in active frontline zones, all empirical artifacts—including session transcripts, consultative logs, and institutional records—were fully anonymized during initial data preparation, removing personal identifiers, specific school numbers, and precise geographic locations.

Because empirical data were generated within an ongoing frontline support network during active conflict, raw qualitative transcripts and internal consultative repositories cannot be publicly shared due to critical participant safety and security protocols (Powell et al., 2025; World Health Organization UNICEF, 2024). Analytical priorities focused exclusively on examining processes of collective knowledge mobilization, professional learning, and sustained accompaniment, rather than evaluating clinical treatment efficacy or individual psychological outcomes (Inter-Agency Network for Education in Emergencies, 2024; Tol et al., 2023).

## Results

### Evolution of professional concerns during protracted war

Longitudinal thematic analysis of the empirical corpus (*N* = 1,248 units) demonstrates a systemic shift in professional concerns across the 2023–2026 period. During the initial establishment of the laboratory (2023), practitioner inquiries primarily reflected acute crisis responses. Early thematic clusters centered on immediate psychological stabilization, acute stress protocols, crisis adaptation, and safety management for displaced or shelled educational communities (Burde et al., 2017).

As frontline conflict conditions persisted, professional requests transitioned from acute trauma management toward addressing the cumulative impacts of continuous traumatic stress and shared traumatic reality (Atli and Çitil Akyol, 2026; Paryente and Frei-Landau, 2024). Educational practitioners—operating simultaneously as affected individuals and “wounded healers”—increasingly documented complex challenges related to ambiguous loss, professional burnout, digital fatigue, and declining student motivation during prolonged distance learning (Powell et al., 2025). Importantly, all student-related observations within the dataset reflect indirect pedagogical evaluations and psychological reporting by adult practitioners rather than direct child assessments.

This longitudinal trajectory indicates a transition from short-term crisis containment to sustained, systemic adaptation. The central practitioner concern shifted from immediate stabilization (“How to manage acute shock?”) toward operational continuity (“How to sustain educational accompaniment under chronic threat?”). Data analysis across the corpus yielded four core thematic domains.

### Psychological stability and emotional wellbeing

The first empirical domain centers on psychological stability and emotional wellbeing under chronic wartime duress. Across the text corpus, practitioners documented persistent emotional fatigue, elevated anxiety, and reduced stress tolerance over multi-year exposure to frontline threats. Analysis revealed a clear conceptual distinction made by practitioners between acute trauma responses and the cumulative impact of prolonged stress. Over time, laboratory deliberations transitioned from immediate crisis management to structural strategies for sustaining basic psychological functioning.

This trajectory highlighted the phenomenon of the “wounded healer” and shared traumatic reality, wherein educational practitioners provided psychosocial support while navigating identical physical threats and ambiguous loss alongside their students (Atli and Çitil Akyol, 2026; Paryente and Frei-Landau, 2024). For instance, practitioner logs reflected acute professional strain and the need for sustained peer reflection:

“*It is increasingly difficult to remain a stabilizing anchor for children when we experience the same shelling and power outages. We need systematic spaces not just to learn techniques, but to process our own secondary exhaustion.”* (School Psychologist, 2024, Ref-CR17)

Consequently, inquiries regarding emotional self-regulation and peer support mechanisms became central to the laboratory's operational schedule, illustrating a shift from isolated crisis responses toward continuous professional accompaniment (Tol et al., 2023; World Health Organization UNICEF, 2024).

### Educational participation and motivation

The second empirical domain addresses the erosion of student educational engagement and academic motivation under persistent crisis conditions. Practitioners systematically documented declining concentration spans, reduced learning persistence, and difficulties maintaining academic goals among students in frontline communities. Rather than viewing learning loss purely as an instructional or curricular deficit, laboratory deliberations increasingly re-framed disengagement as a primary behavioral manifestation of chronic psychological distress and cognitive overload.

This shift reflected the adoption of a trauma-informed pedagogical perspective, wherein sudden academic withdrawal or camera disengagement during online classes was evaluated as an adaptive stress response requiring psychosocial stabilization prior to instructional remediation. Operational consultation logs highlighted this daily pedagogical challenge:

“*When children stop submitting assignments or turn off their cameras, it is rarely passive resistance; it is cognitive exhaustion from nighttime shelling. We had to train teachers not to punish missed deadlines, but to initiate trauma-informed check-ins.”* (General Educator, 2024, Ref-EDU09)

This conceptual transition aligns with international frameworks emphasizing embedded mental health monitoring and flexible learning pathways within crisis-affected educational ecosystems [UNESCO, 2024; UN Children's Fund (UNICEF), 2024; World Bank, 2022].

### Digital overload and distance-learning adaptation

The third empirical domain addresses the psychological and developmental consequences of prolonged reliance on digital learning environments. Educational practitioners systematically documented digital overload and screen fatigue across students, families, and teaching staff. While initial deliberations in 2022 focused on technical infrastructure and connectivity, later records (2024–2026) shifted toward analyzing the socio-emotional deficits stemming from sustained virtual schooling, including reduced interpersonal engagement, social withdrawal, and screen-induced cognitive fatigue.

Educator and parent consultations highlighted that prolonged safety-induced isolation altered classroom dynamics, where passive camera disengagement often signaled socio-emotional withdrawal rather than technical non-compliance. An operational entry from a primary educator illustrates this reality:

“*Distance learning is no longer about setting up Zoom links; it is about reaching children who have spent years behind turned-off cameras. Without deliberate spaces for peer interaction, students lose basic social communication skills, and teachers experience severe emotional exhaustion trying to teach to blank screens.”* (Primary Educator, 2024, Ref-EDU14)

To counteract these socio-emotional deficits, laboratory activities increasingly focused on designing compensatory pedagogical strategies—such as low-bandwidth asynchronous tasks, peer-mentoring circles, and non-digital creative expression—aligning with international emergency education recommendations on mitigating the long-term impact of remote teaching (Inter-Agency Network for Education in Emergencies, 2024; Thomas et al., 2019).

### Support capacity of educational professionals

The fourth empirical domain examines the psychological wellbeing and systemic support capacity of educational professionals working within a shared traumatic reality. School psychologists, classroom teachers, and educational leaders consistently documented severe emotional exhaustion and professional overload. Practitioners faced the dual burden of providing psychosocial stabilization to vulnerable students while simultaneously managing their own exposure to ongoing military threat and displacement—a dynamic reflecting the “wounded healer” phenomenon.

As conflict protracted, laboratory deliberations established that institutional support for children cannot be operationalized independently of the mental health of frontline educators. Consequently, peer consultation logs recorded a substantial rise in inquiries regarding personal resilience, secondary traumatic stress, and burnout prevention. A practitioner reflection illustrates this structural vulnerability:

“*We spent the first year focused entirely on saving the children, but by 2025, the teachers themselves were completely drained. You cannot give emotional support from an empty cup. If the system does not protect the psychologist and the classroom teacher, the entire educational network collapses.”* **(**School Psychologist/Peer Mentor, 2025, Ref-PSYC12)

This focus on workforce capacity underscores that safeguarding educator wellbeing is an essential operational baseline for sustaining crisis-affected educational ecosystems (Inter-Agency Network for Education in Emergencies, 2024; Paryente and Frei-Landau, 2024; Tol et al., 2023; World Health Organization UNICEF, 2024).

### Longitudinal trajectory of practitioner requests

Analysis of the longitudinal text corpus (2023–2026) demonstrates an empirical shift from acute crisis management toward sustained educational adaptation. This trajectory tracks the operational evolution of regional schooling as wartime conditions transitioned from episodic emergency disruptions into an ongoing operational environment.

### From professional requests to practical responses: the knowledge mobilization model

A central empirical finding is that the laboratory functioned as a institutional knowledge mobilization mechanism through which emerging practitioner concerns were processed and translated into actionable pedagogical resources. Rather than remaining isolated queries, practitioner inquiries initiated collaborative cycles of peer reflection, interdisciplinary consultation, and rapid tool adaptation.

Qualitative tracking revealed three operational features characterizing this knowledge mobilization process:

- Collective reframing of individual isolation: frontline practitioner concerns were systematically reframed as shared operational challenges. Case anomalies initially raised by individual school psychologists provided the analytical foundation for regional peer consultations across multiple institutions and municipal stakeholders (DuFour, 2004; Stoll et al., 2006).
- Ecological target alignment: practitioner guidance avoided narrow child-remediation models, focusing instead on the broader ecological layers surrounding the student—specifically virtual learning environments, home–school communication channels, and peer support structures (Masten and Motti-Stefanidi, 2020; Ungar, 2011).
- Prioritization of temporal sustainability: developed tools prioritized multi-year operational continuity over short-term emergency fixes, enabling educational networks to sustain core pedagogical functions during protracted disruption (Damschroder et al., 2009; Inter-Agency Network for Education in Emergencies, 2024).

### The five-stage knowledge mobilization sequence

The transition of raw queries into pedagogical practice operated through a cyclical, horizontal knowledge mobilization pipeline rather than a top-down training framework. Across distinct thematic domains—including acute anxiety, student disengagement, screen fatigue, and educator burnout—data analysis identified a recurring five-stage sequence:

Emerging challenge → Professional request → Collective reflection → Practical recommendation → Educational application

The structural role of each stage within this operational pipeline is defined as follows:

1. Emerging challenge: unmapped educational or psychosocial difficulties surface on the frontlines of wartime instruction (e.g., severe camera disengagement during active shelling). Due to the novel nature of prolonged conflict, standard pedagogical guidelines prove insufficient.
2. Professional request: frontline practitioners (school psychologists, educators, or school leaders) formulate practice-grounded queries and submit them directly to the laboratory as structured agenda items.
3. Collective reflection: the laboratory mobilizes its interdisciplinary network to conduct synchronous consultative sessions. Psychologists, social pedagogues, and administrators collaboratively analyze the request, triangulating field observations with professional literature.
4. Practical/Methodological recommendation: insights are synthesized into structured, actionable methodological artifacts, including regional recommendation volumes and contextualized guidance sheets.
5. Educational application: co-created materials are circulated across the regional school network via digital platforms, enabling practitioners to implement adapted strategies within virtual or hybrid learning environments.

While this five-stage sequence demonstrates consistent structural logic across the text corpus, field application was not entirely linear. The pipeline incorporated essential iterative feedback loops, where practical implementation at Stage 5 routinely generated secondary requests (Stage 2) regarding localized implementation friction, resource constraints, or acute staff burnout. By maintaining horizontal feedback rather than issuing rigid top-down mandates, the laboratory operationalized organic professional learning under conditions of continuous environmental stress (DuFour, 2004; Levin, 2013; Ward, 2017).

### From rehabilitation to resilience: shifting orientations in professional discourse

Analysis of the longitudinal text corpus reveals a profound conceptual reorientation of professional discourse over time. When established in mid-2023, the laboratory's initial operational focus prioritized acute trauma triage and immediate psychological stabilization under active martial law. Early logs reflected a reactive, clinical orientation heavily saturated with terms related to acute stress reactions, emergency emotional stabilization, and immediate grief accompaniment.

However, empirical records from the 2024 to 2026 period demonstrate a structural shift toward proactive, resource-oriented models of systemic resilience and adaptive continuity. Rather than framing interventions solely around post-event trauma recovery, practitioners increasingly focused on sustaining educational and psychological wellbeing within a state of prolonged, active uncertainty. Longitudinal tracking of psychological service registries across this period established clear role-differentiated inquiry dynamics:

- Student dynamics (as reported by educators and parents): initial acute panic during shelling, fear of loss, and relocation anxiety (2022–2023) gradually evolved into chronic manifestations by 2024–2026, including apathy, severe attention deficits, sleep disturbances, and somatization. Prolonged safety-induced isolation elevated gadget dependence, eroding interpersonal interaction skills.
- Parental inquiries: requests shifted from emergency displacement advice and acute loss notification toward addressing severe psycho-emotional depletion among parents—particularly single mothers—who reported an absence of internal resources while coping with children's panic attacks, night terrors, and obsessive tics.
- Educator inquiries: teacher requests transitioned from initial technical adaptations to remote instruction toward personal professional burnout prevention, emotional self-regulation under chronic shelling, and managing classroom apathy or disengagement.

A representative consultation log illustrates this longitudinal transformation:

“*In 2023, our inquiries focused on acute grief and survival panic during air raids. By 2025, the primary challenge became chronic emotional depletion—parents have no internal reserve left to anchor their children, and teachers are operating under severe secondary burnout.”* (School Psychologist Log, 2025, Ref-PSYC08)

This conceptual shift does not imply that war-related stressors subsided; rather, it reflects a collective realization that educational communities require sustainable frameworks designed for living inside an ongoing crisis rather than recovering from a completed one (Inter-Agency Network for Education in Emergencies, 2024; Paryente and Frei-Landau, 2024; Tol et al., 2023).

### The Collective Educational and Psychological Adaptation Model (CEPAM)

**Figure 1:**
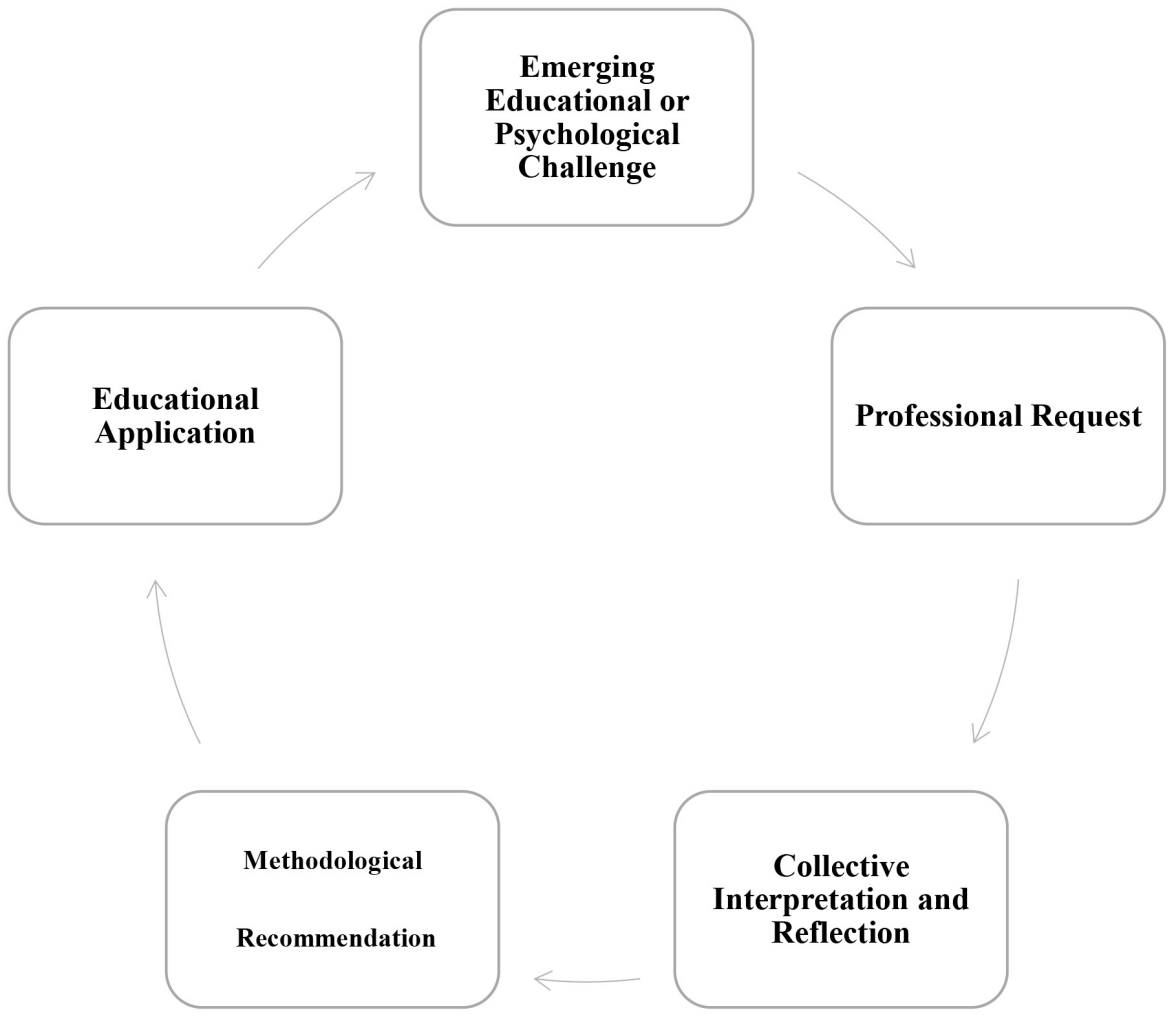
The Collective Educational and Psychological Adaptation Model (CEPAM).

Empirical analysis of the structural relationships among incoming practitioner requests, peer deliberations, expert consultations, co-created recommendations, and field applications reveals a self-regulating institutional loop. This process is conceptualized as the Collective Educational and Psychological Adaptation Model (CEPAM). Distinct from conventional Professional Learning Communities (PLCs), CEPAM functions specifically as an emergency knowledge mobilization framework operating under conditions of active military disruption. This process is conceptualized as the Collective Educational and Psychological Adaptation Model (CEPAM) (Figure 1).

The CEPAM framework highlights four key operational characteristics defining regional adaptation:

- Frontline early identification: practitioners act as first-line detectors of emerging psychological anomalies. Analysis indicates that student and teacher distress became visible in field logs months before appearing in centralized administrative guidance.
- Collective interpretation: horizontal peer reflection transforms fragmented individual cases into a shared regional understanding, mitigating practitioner isolation and enabling coordinated response strategies (DuFour, 2004; Stoll et al., 2006).
- Endogenous knowledge generation: rather than relying solely on imported external theories, local educational communities actively co-create, refine, and validate contextually grounded pedagogical tools (Levin, 2013; Ward, 2017).
- Recursive feedback adaptation: systemic adaptation operates recursively. Rather than following a linear trajectory, practical application routinely generates secondary implementation friction, resource constraints, and staff exhaustion, triggering subsequent cycles of model refinement (Damschroder et al., 2009).

Systemic resilience in prolonged crisis depends fundamentally on the presence of functional, horizontal mechanisms capable of processing raw operational strain into structured collective practices (Masten and Motti-Stefanidi, 2020; Ungar, 2011; World Health Organization UNICEF, 2024).

### Distribution of empirical content across request clusters

To establish the thematic density of the processed text corpus (*N* = 1,248), structural codes were aggregated into nine core thematic clusters. Table 3 illustrates the relative frequency and analytical focus of these request domains across the 2023–2026 period.

**Table 3:** Thematic distribution and relative frequency of professional request clusters (*N* = 1,248 corpus units).

Corpus domain/Professional request cluster	Relative frequency (%)	Primary analytical focus across text records
Psychological rehabilitation and stress concerns	24.2	Acute trauma response, emotional stabilization, panic mitigation, and acute displacement anxiety
Educational atmosphere and learning participation	22.1	Chronic student disengagement, loss of academic interest, passive withdrawal, and trauma-informed pedagogy
Family support and family–school cooperation	14.5	Parental emotional depletion, single-mother support, aligning domestic routines with online schedules
Teacher and school psychologist burnout	12.8	Secondary traumatic stress, compassion fatigue, and professional stamina degradation under chronic shelling
Motivation and developmental continuity	11.3	Multi-year planning breakdown, loss of future orientation among adolescents, and structural learning gaps
Digital overload and distance-learning fatigue	8.4	Severe screen fatigue, social isolation on virtual platforms, and erosion of micro-social peer interactions
Dual schooling and educational overload	4.6	Navigating conflicting demands of Ukrainian distance learning combined with host-country schooling
Behavioral and disciplinary concerns	1.8	Aggressive stress externalization, micro-conflicts in virtual class chats, and emotional self-regulation lapses
Gender-related concerns	0.3	Shifts in domestic and emotional labor distributions among displaced female educators and mothers

Relative frequency percentages reflect the proportional density of specific structural codes extracted from the total coded text corpus (N = 1,248 specific request logs, transcript segments, and consulting session notes; see Krippendorff, 2019; Saldaña, 2021).

## Discussion

This study examined how the Kherson Educational-Psychological Laboratory operated as an institutional mechanism for collective adaptation during the 2023–2026 period of protracted warfare. By analyzing longitudinal text records, the findings contribute to Education in Emergencies (EiE) literature by shifting analytical focus from individual child trauma toward the institutional mechanisms through which local educational communities mobilize, adapt, and circulate practical responses under active martial law (Burde et al., 2017).

### Beyond intervention: understanding educational accompaniment and systemic response capacity

A central theoretical implication concerns the operational distinction between episodic crisis intervention and continuous educational accompaniment. Contemporary Mental Health and Psychosocial Support (MHPSS) frameworks often emphasize standardized, donor-funded intervention packages targeting acute psychological symptoms (Bangpan et al., 2024; Maynard et al., 2019). While critical during immediate acute phases, the empirical trajectory from Kherson indicates that protracted, multi-year conflict alters operational demands.

As conflict extends across multiple years, educational communities operate within a shared traumatic reality where educators and students simultaneously face ongoing bombardment, displacement, and chronic uncertainty (Paryente and Frei-Landau, 2024). Under these conditions, isolated interventions prove insufficient. Practitioners require institutionalized mechanisms capable of expanding regional response capacity dynamically. Because new pedagogical anomalies continuously emerge while previous stressors persist, sustainable support depends on permanent structures for clinical consultation, peer reflection, and rapid tool adaptation.

The laboratory operationalized this continuous accompaniment framework. Rather than delivering episodic training, it provided a stable professional infrastructure wherein educators, school psychologists, and administrators identified frontline challenges, deliberated their psychological implications, and co-created contextually tailored pedagogical guidance. This finding aligns with global EiE recommendations emphasizing that psychosocial infrastructure should be anchored within permanent, local professional networks to ensure long-term operational sustainability (Inter-Agency Network for Education in Emergencies, 2024; Tol et al., 2023).

### Educational challenges as primary behavioral indicators of psychological distress

A second key finding highlights the systemic relationship between classroom engagement and student psychosocial wellbeing. Across the text corpus, educators and school psychologists repeatedly logged instances of declining academic motivation, attention deficits, and passive withdrawal during virtual instruction. Within the laboratory's analytical framework, these instructional barriers were interpreted not as disciplinary infractions, but as primary observational proxies of underlying cumulative distress.

This empirical observation aligns with socio-ecological frameworks of child development (Bronfenbrenner, 1979) and conservation of resources (COR) theory, which articulate how environmental stressors deplete cognitive and emotional reserves required for academic tasks (Atli and Çitil Akyol, 2026; Hobfoll, 1989). In protracted crisis environments, severe difficulties in processing learning material often reflect unvoiced burdens associated with ongoing shelling, domestic disruption, and social isolation. Tracking daily instructional participation—such as camera engagement, assignment completion, and peer interaction—serves as a non-invasive, frontline observational indicator of student wellbeing.

Framing academic metrics as observational proxies for distress enables frontline educators to identify vulnerable children without relying on invasive clinical screenings. This operationalization supports contemporary socio-ecological resilience research, demonstrating that maintaining structured educational participation acts as a protective anchor for child developmental trajectories amidst protracted instability (Masten and Motti-Stefanidi, 2020; World Bank, 2022).

### Digital overload as an enfolding crisis in prolonged distance learning

The prominent emergence of digital overload within practitioner inquiry logs highlights a critical operational challenge in multi-year distance education. While emergency remote teaching literature traditionally focuses on technological infrastructure and internet access, these empirical findings indicate that prolonged virtual participation during active conflict generates distinct psychosocial and developmental pressures.

Practitioners routinely logged observational records detailing screen fatigue, diminished attention spans, severe communication barriers, and social isolation among students navigating sustained online instruction under safety threats. Rather than being framed solely as technical friction, prolonged virtual engagement was identified as an ecological stressor that exacerbates relational fragmentation. While digital platforms remain essential for operational continuity and physical safety under active shelling, educational systems must embed socio-emotional countermeasures to mitigate screen-induced fatigue and domestic isolation.

These observations demonstrate that emergency remote pedagogy requires a shift from purely technology-centric optimization toward relationally anchored learning frameworks. Without structured psychosocial support integrated directly into virtual routines, prolonged platform dependence inadvertently compounds the relational vulnerabilities of children and educators in protracted crisis settings [UNESCO, 2024; UN Children's Fund (UNICEF), 2024].

### Educational communities as endogenous knowledge-producing systems

The findings offer empirical insights into contemporary knowledge mobilization research in high-stress operational environments. Traditional institutional frameworks often conceptualize professional knowledge as an asset generated through centralized research and subsequently disseminated top-down into field practice. While top-down guidance remains necessary, these findings illustrate an endogenous knowledge mobilization mechanism wherein practical pedagogical guidance emerges horizontally from frontline operational experience.

Within the laboratory's consultative structure, practice-grounded inquiries raised by school psychologists, classroom teachers, social pedagogues, and administrators served as the catalyst for peer deliberation. Through structured interdisciplinary consultation, localized responses were synthesized and circulated across the regional educational network. These empirical patterns align with knowledge mobilization theories (Cooper et al., 2009; Levin, 2013; Ward, 2017), demonstrating that frontline practitioners act as active co-creators of contextualized pedagogical tools.

In protracted crisis settings, horizontal knowledge production becomes critical because frontline disruptions evolve faster than centralized administrative guidance can be generated. Distinct from conventional professional learning communities operating under stable conditions, this horizontal loop functions under active martial law as an adaptive mechanism, converting raw operational strain into context-specific pedagogical support (DuFour, 2004; Stoll et al., 2006).

### Reconsidering resilience in educational contexts as a systemic process

The findings contribute to international scholarship surrounding the conceptualization of resilience in conflict-affected education. Psychological literature frequently emphasizes individual adaptive capacities, cognitive traits, and internal protective factors that support developmental trajectories amidst adversity (Masten, 2001; Ungar, 2011). While acknowledging these foundational perspectives, these longitudinal findings indicate that under conditions of multi-year warfare, resilience must also be conceptualized as a relational, environmental, and institutional process.

Throughout the text corpus, resilience was rarely framed by practitioners as an isolated individual trait. Instead, professional discourse consistently highlighted the role of supportive interpersonal relationships, emotional safety within virtual spaces, adult relational anchoring, open family–school communication, and institutional support networks. Rather than locating resilience solely within the child, frontline practices focused on constructing protective ecological buffers.

This consensus aligns with socio-ecological models of systemic resilience (Masten and Motti-Stefanidi, 2020; Ungar, 2011). It demonstrates that educational communities—when provided with horizontal consultation mechanisms—act as resilience-generating micro-systems. The capacity of regional networks, schools, and multidisciplinary teams to adapt collectively, exchange field data, and co-create context-specific tools represents a vital dimension of systemic survival. Anchoring resilience within collective professional layer shifts intervention focus from individual vulnerability toward the structural durability of the educational environment (Inter-Agency Network for Education in Emergencies, 2024; World Health Organization UNICEF, 2024).

### Theoretical and practical contributions of the CEPAM framework

The Collective Educational and Psychological Adaptation Model (CEPAM) provides a conceptual framework for understanding systemic adaptation loops in conflict-affected educational systems. The model theorizes regional adaptation not as a static top-down mandate, but as a dynamic, recursive pipeline connecting frontline challenges, practitioner inquiries, collective reflection, endogenous tool co-creation, and network-wide application.

While established international MHPSS frameworks frequently prioritize individual diagnostic tracking or standardized clinical interventions, CEPAM complements these approaches by capturing the horizontal, collective processes operating within professional networks. Consequently, this framework contributes to the literature in three key ways:

- Advancing Education in Emergencies (EiE) Research: it shifts analytical focus from documenting static crisis outcomes (e.g., learning loss or symptom prevalence) toward analyzing institutional mechanisms of adaptive capacity (Burde et al., 2017).
- Delineating Emergency Professional Learning Communities (PLCs): distinct from conventional PLCs operating under stable organizational conditions, CEPAM demonstrates how professional networks function as emergency knowledge mobilization hubs under active martial law, chronic shelling, and infrastructure disruption (DuFour, 2004; Stoll et al., 2006).
- Enriching knowledge mobilization frameworks: it illustrates how actionable pedagogical guidance can be co-created directly from frontline operational queries and circulated through regional digital channels (Levin, 2013; Ward, 2017).

While developed within the context of wartime education in the Kherson region, the structural architecture of CEPAM offers adaptable insights for other education systems facing active military conflict, forced displacement, or prolonged systemic disruption (Inter-Agency Network for Education in Emergencies, 2024).

### The longitudinal evolution of frontline concerns (2023–2026)

To trace how operational priorities shifted as the military conflict transformed from an acute emergency into a protracted crisis, the text corpus (*N* = 1,248) was analyzed chronologically (Saldaña, 2021). Table 4 maps this empirical evolution, documenting the longitudinal transformation of professional inquiries and laboratory support strategies across the 2023–2026 period.

**Table 4:** Evolution of professional concerns and support focus during protracted war (2023–2026).

Period	Dominant frontline professional concerns	Characteristic focus of laboratory support
2023	Acute stress reactions, emergency physical safety threats, relocation panic, and immediate grief accompaniment	Immediate crisis triage, emotional stabilization, and psychological first aid under active martial law
2024	Family displacement strain, severe parental depletion, erosion of classroom atmosphere, and coping with prolonged uncertainty	Strengthening family–school cooperation, rebuilding predictable routines, and supporting parental coping capacity
2025	Chronic digital overload, distance-learning fatigue, academic disengagement, and dual-schooling friction	Sustaining instructional participation, mitigating virtual fatigue, and addressing dual-curriculum overload
2026	Systemic professional burnout, secondary traumatic stress, loss of adolescent future orientation, and long-term sustainability	Institutional capacity expansion, burnout prevention, maintaining practitioner stamina, and developmental continuity

Categories were derived through thematic coding of longitudinal seminar logs, consultation transcripts, and methodological records (N = 1,248). Concerns reflect practitioner observations across student, parent, and educator stakeholder groups (Krippendorff, 2019; Saldaña, 2021).

This longitudinal trajectory demonstrates a structural evolution in operational priorities. Early institutional efforts in 2023 focused strictly on immediate stabilization and acute emergency response. By 2025–2026, professional inquiries transitioned toward managing chronic emotional depletion, professional burnout, digital fatigue, and systemic institutional stamina. These findings illustrate that protracted conflict fundamentally alters both the nature of educational vulnerabilities and the institutional support mechanisms required by frontline communities (Masten and Motti-Stefanidi, 2020; World Bank, 2022).

### Practical implications for policy and practice

The empirical synthesis of longitudinal laboratory records (*N* = 1,248) yields four core structural implications for educational policymakers, international development agencies, and frontline school administrators operating in active conflict zones:

- Institutionalizing decentralized support infrastructure: educational systems during protracted crises require decentralized, regional mechanisms capable of capturing real-time frontline inquiries, facilitating peer-to-peer expert consultation, and rapidly disseminating context-sensitive methodological guidance (Inter-Agency Network for Education in Emergencies, 2024). Decentralized nodes reduce administrative latency and ensure operational responsiveness when centralized channels face disruption.
- Prioritizing adult wellbeing as a prerequisite for student resilience: psychological and pedagogical interventions aimed at child welfare are structurally interdependent with the institutional support provided to educators, school psychologists, and caregivers. Sustaining the operational capacity of frontline education requires systematic burnout monitoring, secondary traumatic stress mitigations, and targeted professional accompaniment for adult anchors (Bangpan et al., 2024; Maynard et al., 2019).
- Transitioning from passive resource delivery to knowledge transformation: sustainable educational adaptation under active military conditions requires more than static material assistance or rigid standard operating procedures. Institutional structures must establish iterative feedback loops capable of converting localized crisis management experiences into validated, scalable professional practices (Levin, 2013).
- Leveraging low-cost micro-institutional structures: modest, regional consultative entities can provide substantial systemic support during extended emergencies. Their primary institutional value lies in maintaining unbroken operational continuity, providing predictable professional reflection spaces, and supporting collaborative adaptation across frontline educational networks (World Bank, 2022).

Ultimately, maintaining educational functionality and psychological support during protracted war depends not only on existing institutional assets, but fundamentally on an educational network's capacity for continuous learning, systematic feedback integration, and adaptive professional collaboration.

### Operational matrix of wartime challenges and systemic responses

The practical utility of the CEPAM framework lies in its iterative capacity to transform frontline practitioner concerns into operationalized institutional strategies. Rather than treating adaptation as a static crisis intervention, the model coordinates a continuous feedback loop through which regional educational communities identify emerging wartime anomalies, translate practitioner inquiries into structured professional knowledge, and redistribute context-sensitive methodological tools back through school networks (Damschroder et al., 2009).

To illustrate how this adaptive cycle operated in practice across the 2023–2026 period, Table 5 synthesizes the major systemic challenges logged within the text corpus (*N* = 1,248) alongside their frontline behavioral expressions and the dominant pedagogical and MHPSS strategies co-created to mitigate them.

**Table 5:** Matrix of major wartime challenges, typical professional concerns, and dominant educational responses.

Core systemic challenge	Typical frontline professional concern	Dominant educational and psychosocial response
Chronic anxiety and uncertainty	Acute emotional instability, panic episodes, and reduced psychological coping capacity observed among students	Implementation of emotional self-regulation techniques, stabilization of daily virtual classroom routines, and targeted resilience-building activities
Declining educational motivation	Progressive academic disengagement, passive camera/screen withdrawal, and loss of learning purpose	Strengthening active classroom participation methods, introducing educational gamification, and infusing learning activities with relational meaning
Sustained digital overload	Screen fatigue, diminished attention spans, and communication barriers in multi-year distance learning	Balancing virtual instruction with offline experiential tasks, structured micro-breaks, and creative non-digital expression
Dual-schooling pressures	Academic overload and conflicting curriculum expectations faced by displaced students navigating domestic and host systems	Flexible academic scheduling, customized workload adjustments, and direct family–school consultations to reconcile dual curriculum demands
Teacher and psychologist burnout	Secondary traumatic stress, emotional exhaustion, and diminished instructional stamina among educational staff	Institutionalizing structured peer-support groups, psychological decompression sessions, and collaborative professional reflection spaces
Compounded family stress	Fragmented domestic support environments resulting from displacement, economic strain, and ongoing wartime volatility	Enhancing family–school coordination, providing targeted parental psychological guidance, and establishing virtual school-community support hubs

Categories and response domains were synthesized from longitudinal corpus records (N = 1,248) and validated against established international frameworks for trauma-informed pedagogy, educator wellbeing, and institutional support in emergency education settings (Brunzell et al., 2015; Henderson, 2023; Teacher Task Force, 2019; Thomas et al., 2019).

## Limitations of the study

Several methodological and contextual limitations should be considered when interpreting these findings:

- Regional and contextual specificity: this research represents a single qualitative case study situated within the high-intensity wartime context of the Kherson region, Ukraine. While providing longitudinal insights into adaptation processes during active conflict, the findings are not intended to be statistically generalizable to all emergency education settings globally (United Nations Office for the Coordination of Humanitarian Affairs, 2025, UNESCO, 2025).
- Observational proxy data (lack of direct child-level metrics): the text corpus (*N* = 1,248) reflects professional communications, consultation logs, and strategic inquiries among adult educational actors (educators, school psychologists, administrators). Consequently, student behavioral and emotional dynamics are captured through practitioner observations and professional requests rather than direct child psychometric assessments or clinical outcome measures.
- Non-experimental design: the analyzed corpus comprises naturally occurring practice-grounded Professional records rather than experimental or controlled intervention data. While this approach preserves ecological validity by capturing authentic frontline inquiries, it precludes strict causal inferences regarding the isolated efficacy of specific pedagogical tools (Krippendorff, 2019).
- Self-selection bias: participation in the laboratory's consultative network was voluntary. The corpus primarily reflects insights from professionals motivated to engage in horizontal peer consultation and continuous professional reflection, which may not fully represent non-participating institutions within the broader educational system.
- Variability in implementation fidelity: evidence regarding field implementation was derived from documented consultations, methodological artifacts, and practitioner feedback. The corpus structure does not permit empirical measurement of implementation fidelity or uniform application across all participating schools.
- Evolving conflict dynamics: data collection occurred during active and fluid military conditions. As wartime conditions evolve, institutional priorities and support needs will continue to shift dynamically (UNESCO, 2023).

Despite these boundaries, the longitudinal corpus provides a systematic record of collective professional adaptation under active conflict, contributing empirical evidence to the literature on institutional resilience in Education in Emergencies.

## Conclusion

This study examined how a regional Educational-Psychological Laboratory in the Kherson region of Ukraine functioned as an institutional mechanism of collective educational and psychological adaptation during the protracted 2023–2026 conflict. Rather than evaluating individual student-level outcomes, the investigation analyzed how frontline educational professionals identified emerging wartime challenges, interpreted their systemic implications, co-created practical pedagogical responses, and redistributed methodological guidance through regional networks (*N* = 1,248). In doing so, this research shifts analytical focus in Education in Emergencies toward decoding the institutional processes through which local educational communities sustain their internal support capacity during prolonged crisis conditions.

Longitudinal analysis of the multi-year text corpus—comprising inquiry logs, seminar transcripts, consultation records, and methodological materials—demonstrated that wartime disruptions were rarely interpreted by practitioners as isolated psychological problems. Instead, professional discourse consistently framed child wellbeing as structurally interdependent with instructional participation, family stability, virtual classroom climate, adult coping capacity, and developmental continuity (World Health Organization UNICEF, 2024).

Furthermore, the findings document a distinct temporal evolution in professional priorities. While early laboratory activities in 2023 focused on acute psychological stabilization, emergency triage, and crisis response, professional inquiries in 2025 and 2026 shifted toward systemic resilience, digital overload mitigation, educator burnout prevention, and long-term institutional stamina. This transition illustrates that protracted conflict alters both the nature of educational vulnerabilities and the institutional support mechanisms required by frontline practitioners.

Based on these empirical insights, the Collective Educational and Psychological Adaptation Model (CEPAM) conceptualizes regional adaptation as a recursive pipeline connecting frontline challenges, practitioner inquiries, structured peer interpretation, endogenous knowledge co-creation, formal guidance, and renewed classroom application [UN Children's Fund (UNICEF), 2024].

Ultimately, this study contributes to international scholarship in three principal ways:

- Advancing Education in Emergencies (EiE) frameworks: it moves beyond documenting static crisis metrics to analyze the horizontal institutional mechanisms through which local educational communities generate and operationalize practical responses.
- Enriching knowledge mobilization literature: it illustrates how actionable professional knowledge emerges directly from frontline operational experience and circulates through collaborative regional networks.
- Reconceptualizing systemic adaptation: it provides a framework that reframes educational adaptation as a collective, institutional process rather than an isolated individual outcome.

The primary policy implication of this research is that sustaining educational and psychological support during protracted war requires flexible professional micro-structures capable of converting frontline operational challenges into shared pedagogical knowledge. As educational systems globally face ongoing conflict, displacement, and systemic disruption, understanding how frontline communities build and maintain adaptive support structures remains a vital priority for international educational research, policy, and practice.

## Data Availability

The raw data supporting the conclusions of this article will be made available by the authors, without undue reservation.
