# Supplementary material for: Sustaining educational and psychological support during protracted war: a regional laboratory model from Ukraine
Source: Front Psychol. 2026 Aug 25;17:1939975. doi: 10.3389/fpsyg.2026.1939975 (PMC13549876; doi:10.3389/fpsyg.2026.1939975)
Supplement: Supplementary file 1 [file Table_1.docx]

**Supplementary Materials**

**Table S1**

*Analytical Audit Trail: Mapping Raw Practitioner Inquiries to Codes, Categories, and CEPAM Dimensions*

| **Raw Practitioner Inquiry / Text Segment (Excerpt)** | **Initial Inductive Code** | **Sub-Theme / Coded Category** | **CEPAM Model Dimension** |
| --- | --- | --- | --- |
| "Teachers report high fatigue during online classes; children turn off cameras and don't react to tasks." | Camera disengagement; educator fatigue | Digital fatigue and learning motivation loss | Practitioner Request Dynamics (Horizontal Inquiries) |
| "How do we talk to primary students about explosive hazards without causing panic during shelling?" | Safety protocol anxiety; acute stress framing | Crisis communication and physical safety protocols | Practitioner Request Dynamics (Acute Safety Needs) |
| "Psychologists need a space to reflect on their own secondary trauma while supporting displaced families." | Helper burnout; shared traumatic reality | Wounded healer support and peer supervision | Sustained Accompaniment (Peer Consultation & Reflection) |
| "Drafted practical guidelines for educators on recognizing acute trauma signs during distance learning." | Actionable advice generation; peer knowledge sharing | Methodological adaptation and practice guidance | Institutional Knowledge Mobilization (Actionable Recommendations) |

### Table S2

*Thematic Transformation and Longitudinal Coding Matrix of Psychological Service Inquiries Across Educational Stakeholders (2022–2026)*

| **Raw Practitioner Inquiry / Text Segment (Excerpt)** | **Initial Inductive Code** | **Sub-Theme / Coded Category** | **CEPAM Model Dimension** |
| --- | --- | --- | --- |
| "In the early years of the full-scale war, a sharp shift occurred toward concerns regarding persistent air raid alerts, shelling, prolonged distance learning, displacement, loss, and fear of death or sirens." | Crisis reaction; acute war exposure; displacement stress | Acute trauma response & security adaptation | Practitioner Request Dynamics (Acute Safety & Crisis Needs) |
| "In the last two years, students increasingly report chronic fatigue, apathy, impaired concentration, loss of motivation, panic attacks, and severe emotional tension." | Chronic exhaustion; learning demotivation; somatic panic | Prolonged stress manifestations & emotional depletion | Sustained Accompaniment (Individual Psychological Health) |
| "Parents sought advice on child adaptation to changed study/living locations, supporting acute grief reactions, panic states during shelling, dark/noise fears, sleep disturbances, and tics." | Displaced adaptation; acute grief support; somatization | Parental psychoeducation & crisis child support | Systemic Family & Child Support (Acute Interventions) |
| "Children spend excessive time isolated with gadgets, showing growing signs of device addiction and minor delays in psychomotor and speech development." | Digital dependency; social isolation; developmental lag | Screen-time impact & social-communicative deficits | Developmental & Behavioral Monitoring |
| "A growing number of requests come from mothers raising children alone regarding severe psychoemotional exhaustion and lack of internal resources to support their child." | Single-parent overload; resource depletion; maternal burnout | Parental emotional exhaustion & caregiver burnout | Sustained Accompaniment (Parental Wellbeing & Resources) |
| "Initial teacher requests focused on organizing distance learning under martial law, maintaining student attention through screens, and inclusive remote teaching methods." | Remote instruction adaptation; digital engagement | Methodological adaptation in crisis education | Institutional Knowledge Mobilization (Pedagogical Strategies) |
| "Group consultations and practical sessions equipped teachers with trauma awareness, Psychological First Aid (PFA), resource recovery techniques, and self-regulation during alerts." | Trauma-informed training; PFA skills; self-regulation | Teacher psychoeducation & psychological first aid | Institutional Capacity Building (Emergency Preparedness) |
| "Requests from educators shifted toward preventing professional burnout, managing classroom interaction under chronic stress, and addressing student apathy, withdrawal, or aggression." | Professional burnout; chronic stress fatigue; disruptive behaviors | Educator wellbeing & classroom crisis management | Institutional Knowledge Mobilization (Burnout Prevention & Management) |
